# Integrated Omics Reveal *Dendrobium nobile* Lindl.’s Anti-Diabetic Mechanisms via Arginine/Proline and Glycerophospholipid Pathways

**DOI:** 10.3390/ph18071061

**Published:** 2025-07-18

**Authors:** Zhibo Wang, Xian Wang, Sifan Guo, Ying Cai, Dandan Xie, Yujuan Wang, Aihua Zhang, Jun Dai, Shi Qiu

**Affiliations:** 1 International Advanced Functional Omics Platform, Scientific Experiment Center, Hainan Engineering Research Center for Biological Sample Resources of Major Diseases, Hainan Medical University, Xueyuan Road 3, Haikou 571199, China; wangzhibo0720@163.com (Z.W.); bancheng@muhn.edu.cn (X.W.); guosifan612@163.com (S.G.); 13504555786@126.com (Y.C.); dr.dan@hainmc.edu.cn (D.X.); aihuatcm@163.com (A.Z.); 2 Graduate School, Heilongjiang University of Chinese Medicine, Harbin 150040, China; 3 Engineering Technology Research Center of Plant Cell Engineering, West AnHui University, Moon Island, Luan 237000, China; wangyujuanwyj@126.com; 4 Hainan Shengrong Technology Co., Ltd., Yangnan Village 18, Haikou 571156, China

**Keywords:** *Dendrobium nobile* Lindl., metabolomics, proteomics, type 2 diabetes mellitus, mechanism

## Abstract

**Background/Objectives**: *Dendrobium nobile* Lindl. (DNL), a traditional dietary supplement, exhibits therapeutic potential for type 2 diabetes mellitus (T2DM), yet its mechanisms remain unclear. **Methods**: T2DM was induced in *db*/*db* mice. DNL (10 g/kg/d) or metformin (65 mg/kg/d) was administered for 4 weeks. This study integrated pharmacodynamic evaluation and multi-omics to elucidate DNL’s anti-diabetic effects in *db*/*db* mice. **Results**: DNL intervention significantly ameliorated T2DM phenotypes, reducing hyperglycemia, insulin resistance, and renal dysfunction. Metabolomics analysis identified 39 differential metabolites (19 upregulated, 20 downregulated) linked to citrate cycle, oxidative phosphorylation, and glycerophospholipid metabolism, while proteomics revealed 113 differentially expressed proteins, with multi-omics integration highlighting DNL’s modulation of three proteins (Ckm, Ache, Selenbp1) and four metabolites (4-guanidinobutanoic acid, phosphorylcholine, homocysteine, succinic acid) across arginine/proline metabolism, glycerophospholipid metabolism, and sulfur metabolism. Pathway analysis demonstrated DNL’s restoration of dysregulated processes, including inflammation suppression via NF-κB and PI3K-Akt pathways, enhanced insulin sensitivity through glycerophospholipid balance, and mitigation of oxidative stress via sulfur metabolism. Key correlations between metabolites and proteins underscored DNL’s multi-target action. **Conclusions**: These findings systematically decode therapeutic mechanisms of *Dendrobium nobile* Lindl., emphasizing its role in rectifying metabolic disorders and inflammatory signaling, thereby providing a molecular basis for its clinical application in T2DM management.

## 1. Introduction

Type 2 diabetes mellitus (T2DM) is a disease characterized by non-autoimmune heterogeneous progressive pancreatic islet β-cell insulin underproduction [1], which often occurs in the presence of insulin resistance (IR) and the metabolic syndrome (MS) [2,3]. Diabetes has grown up to be a global health burden due to its high morbidity, disability, and mortality. The usual treatment is the implementation of statins and hypoglycaemic agents. In contrast to these applications, which may produce significant side effects, *Dendrobium nobile* Lindl. (DNL) has been demonstrated to generate hypoglycaemic properties without significant side effects [4].

DNL has been used as a dietary additive for centuries [5], and its pharmacological activities are closely related to the chemical components, with pharmacological activities such as anti-tumor, anti-aging, immune enhancement, hypoglycemic, and anti-cataract [6]. Recent phytochemical studies identify key bioactive constituents in DNL, including dendrobine, polysaccharides, and flavonoids, which contribute to its hypoglycemic effects [7,8]. DNL polysaccharides enhance insulin sensitivity via AMPK activation [9], while dendrobine analogues can improve diabetic nephropathy by regulating glucose metabolism [10]. These compounds collectively modulate metabolic pathways relevant to T2DM pathogenesis. Clinically, it can be used to treat patients with metabolic syndrome [11]. However, the unclear mechanisms of DNL’s anti-diabetic action limit its clinical translation. It is imperative to develop a method to elucidate the mechanism of action of DNL.

Metabolomics can capture the panoramic changes of body metabolites through high-throughput technology to reveal the overall regulatory effect of herbs on the biological system of the organism [12]. Metabolomics contributes to the standardized assessment of the efficacy and mechanisms of herbs by providing data-driven, quantifiable evidence of metabolic changes [13]. Proteomics offers a panoramic analysis of the expression and modification of all proteins and their dynamic changes in cells or tissues, a holistic feature that is highly compatible with the systemic mode of action of herbs [14]. Proteomics resolves potential drug targets by means of bioinformatics [15]. The integration of proteomics and metabolomics can achieve the systematic analysis of herbs from phenotype to mechanism, and the construction of a multi-level network to reveal the multi-target mechanism of action of herbs [16].

**Figure 1:**
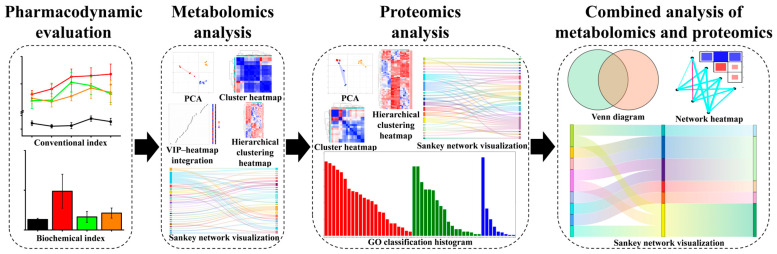
The design path of this study. Firstly, the pharmacodynamics of *db*/*db* mice in each group was studied, including body weight, FBG, HbA1 c level, and pathological observation. Secondly, based on the metabolomics method, the metabolites of DNL callback and their enriched metabolic pathways were screened. Then, based on the proteomics method, the proteins of DNL callback and their enriched pathways were screened. Finally, a combined analysis of metabolomics and proteomics was performed to elucidate the potential mechanism of HGD.

In this study, we used *db*/*db* mice to construct a T2DM mouse model and elucidated the efficacy and mechanism of DNL through experimental examination, metabolomics, and proteomics analyses (Figure 1). The ameliorative effect of DNL on T2DM provides a reference for exploring the indications and mechanism of action of DNL.

## 2. Results

### 2.1. DNL Significantly Attenuated the T2DM Disease Phenotype in db/db Mice

**Figure 2:**
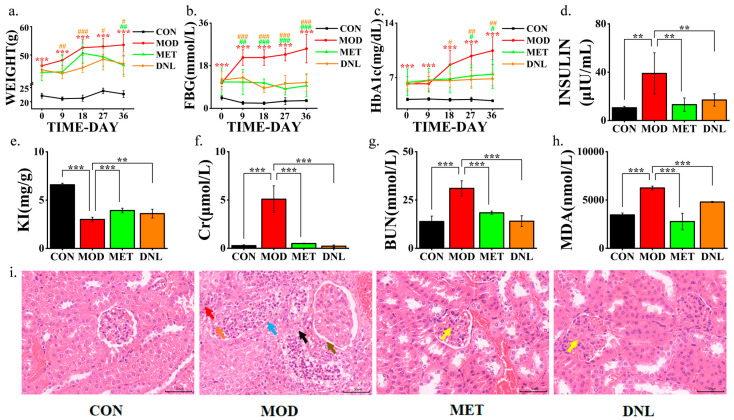
Therapeutic effects of DNL on pathological indicators in *db*/*db* mice with T2DM. (**a**) Body weight dynamics: The model (MOD) group exhibited a progressive increase in body weight over 36 days compared to the control (CON) group, while DNL administration significantly attenuated this trend (# *p* < 0.01 vs. MOD). (**b**) Fasting blood glucose (FBG): MOD mice displayed sustained hyperglycemia, which was markedly reduced by DNL intervention (### *p* < 0.001 vs. MOD). (**c**) Glycated hemoglobin (HbA1c): elevated HbA1c levels in MOD mice were effectively normalized following DNL treatment (## *p* < 0.01 vs. MOD). (**d**) Serum insulin: Hyperinsulinemia in the MOD group was significantly suppressed by DNL (** *p* < 0.01 vs. MOD). (**e**) Kidney index (KI): DNL restored the decreased KI in MOD mice to near-normal levels (** *p* < 0.01 vs. MOD). (**f**) Creatinine (Cr): DNL reversed MOD-induced renal dysfunction, as evidenced by reduced Cr levels (*** *p* < 0.001 vs. MOD). (**g**) Blood urea nitrogen (BUN): Elevated BUN in MOD mice was ameliorated by DNL (*** *p* < 0.001 vs. MOD). (**h**) Malondialdehyde (MDA): Oxidative stress indicated by increased MDA was mitigated by DNL (*** *p* < 0.001 vs. MOD). (**i**) Renal histopathology: H&E staining revealed severe glomerular lesions in MOD mice Neutrophils (orange arrows) Renal tubular epithelial cell necrosis (blue arrows) Proliferation (black arrows) Fibroblasts (red arrows) Basement membrane thickening (brown arrows)), which were alleviated by DNL treatment. Scale bar: 50 μm. Abbreviations: CON, control group; MOD, T2DM model group; MET, metformin-treated group; DNL, *Dendrobium nobile* Lindl.-treated group. Data are expressed as mean ± SEM. Statistical significance: ** *p* < 0.01, *** *p* < 0.001 vs. CON; # *p* < 0.05, ## *p* < 0.01, ### *p* < 0.001 vs. MOD (two-tailed Student′s *t*-test).

To investigate the effects of DNL administration on T2DM, we established a T2DM disease model using *db*/*db* mice. The model exhibited pathophysiological features of T2DM, such as significantly higher body weight (Figure 2a), fasting blood glucose (Figure 2b), glycosylated haemoglobin (Figure 2c), and insulin (Figure 2d) in the MOD group of mice compared to the CON group. In addition, mice in the MOD group had significantly lower renal indices (Figure 2e) compared with the CON group, suggesting renal impairment. Subsequently, we examined the serum levels of Cr, BUN, and MDA in the mice in each group. The results showed that Cr, BUN, and MDA levels were significantly higher in mice in the MOD group compared with the CON group (Figure 2f–h). Observation of H&E staining of renal tissues of mice in each group revealed lesions in the renal tissues of mice in the MOD group (Figure 2i). We treated mice with the drug for 36 days, and salvage was obtained in all the treated groups, which indicates that DNL administration has a significant preventive and curative effect on T2DM mice.

### 2.2. Metabolic Effects of DNL on Serum Profiles of db/db Mice

**Figure 3:**
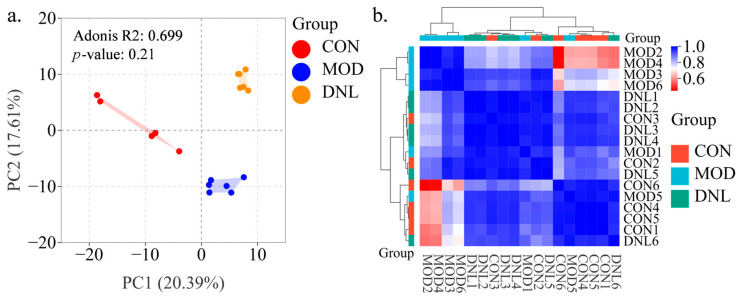
Multi-omics integration reveals DNL-mediated metabolic reprogramming in T2DM mice. (**a**) Principal component analysis (PCA) score plot: Unsupervised multivariate analysis demonstrated distinct clustering patterns among the control (CON), T2DM model (MOD), and DNL-treated groups. Dimensionality reduction via PCA highlighted metabolic trajectory restoration by DNL, with the DNL group positioned intermediately between CON and MOD along principal component 1 (PC1). (**b**) Hierarchical clustering heatmap: Spearman correlation analysis of serum metabolites across groups revealed substance-specific clustering patterns. The MOD group exhibited disrupted metabolic correlations (blue clusters), which were partially restored by DNL intervention (red clusters), indicating coordinated regulation of glycerophospholipid and amino acid metabolism. Abbreviations: CON, control group; MOD, T2DM model group; DNL, *Dendrobium nobile* Lindl.-treated group.

To reveal the metabolites that change after T2DM and DNL administration, we performed non-targeted metabolomics analysis of serum samples from the CON group, the MOD group, and the DNL group. As shown in Figure 3a, the PCA plot showed that the CON group was well separated from the MOD group, and the DNL group had a tendency to deviate from the MOD group and approach the CON group. The correlation clustering analysis showed a clear trend of clustering among the three groups, with the DNL group intermediate between the CON group and the MOD group (Figure 3b).

### 2.3. DNL Exhibited Effects on db/db Mice with T2DM Related Various Metabolites

**Figure 4:**
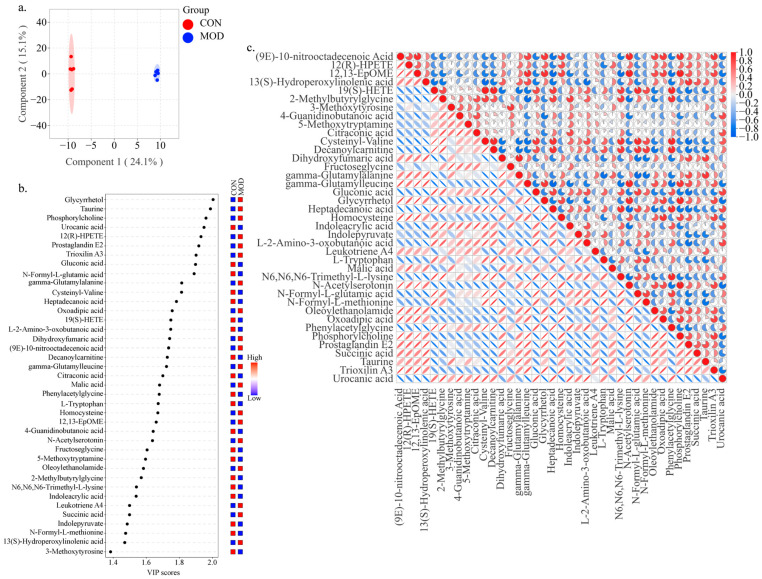

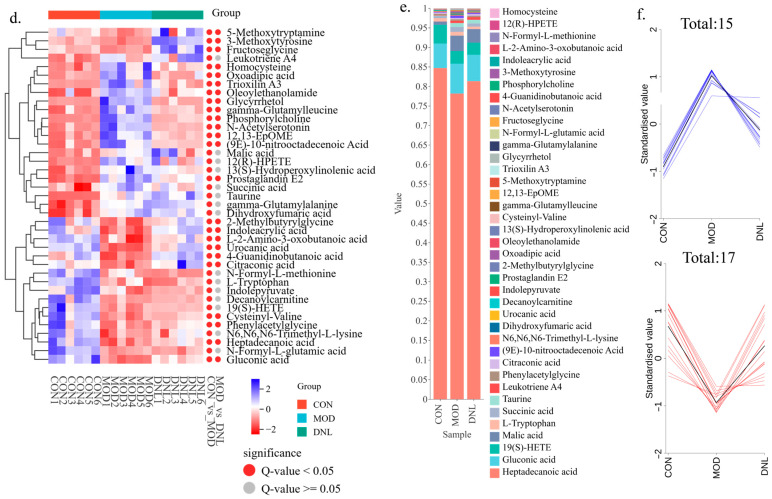
Impact of DNL on metabolic profiles in *db*/*db* mice with T2DM. (**a**). OPLS-DA score plot: Supervised multivariate statistical approach revealing variable importance in projection (VIP) values, enabling preliminary identification of discriminative metabolites between the control (CON) and model (MOD) groups. (**b**). VIP–heatmap integration: Dual visualization combining VIP value distribution and metabolite abundance patterns across CON and MOD groups. (**c**). Correlation heatmap: Inter-metabolite relationship network constructed through pairwise correlation analysis of expression levels in CON and MOD groups. (**d**). Hierarchical clustering heatmap: Color-gradient visualization of metabolite patterns across CON, MOD, and DNL groups, with annotations highlighting significant variations in CON_VS_MOD and MOD_VS_DNL comparisons. (**e**). Stacked bar chart: Group-wise distribution and quantitative comparisons of differential metabolites across all experimental groups. (**f**). *K*-means cluster analysis: Bipartite stratification of upregulated/downregulated metabolites in CON/MOD groups, with DNL intervention recovery patterns.

The endogenous metabolites that change in the serum of *db*/*db* mice with T2DM upon DNL intervention were further investigated. The OPLS-DA analysis was performed to identify metabolites contributing to the separation of the CON group and the MOD group. The score plot showed a clear separation between the two groups, and cross-validation plots generated from 200 alignment tests showed intercepts of R2 = 0.999 and Q2 = 0.85, indicating good reliability and predictability of the respective models (Figure 4a). Filtering the initial selection of metabolites according to the criteria of VIP > 1, *p* < 0.05, we screened to obtain 39 metabolites, of which 19 were upregulated and 20 downregulated in the MOD group (Figure 4b). The correlation analysis showed that differential metabolites with similar abundance clustered together, representing an internal relationship between metabolic proximity and differential metabolites (Figure 4c).

The hierarchical clustering dendrogram showed that the metabolites in the MOD group were significantly separated from both the CON group and the DNL group, while the metabolites in the CON group and the DNL group had a similar trend. And 24 differential metabolites were significantly altered in the DNL group compared to the MOD group (Figure 4d). As shown in Figure 4e, the distribution of differential metabolite contents within and across groups showed significant differences. Further, the *K*-means clustering analysis showed that 32 differential metabolites were reversed by DNL intervention (17 upregulated and 15 downregulated), of which 24 were significantly recovered (13 upregulated and 11 downregulated) (Figure 4f and Figure S1).

### 2.4. DNL Exerted Influences on db/db Mice with T2DM-Related Metabolic Pathways

**Figure 5:**
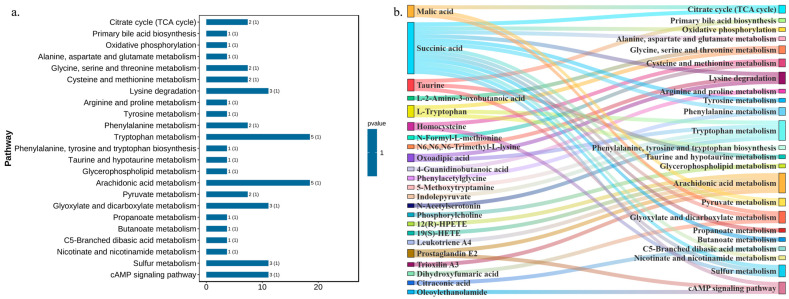
Modulation of T2DM-associated metabolic pathways by DNL in *db*/*db* mice. (**a**). KEGG pathway-enrichment analysis: Bar chart illustrating pathway-specific enrichment of differential metabolites, highlighting key metabolic pathways perturbed in T2DM. (**b**). Sankey network visualization: Flow relationships between enriched metabolic pathways and their corresponding differential metabolites, emphasizing pathway-metabolite connectivity.

Based on the KEGG database, the enrichment analysis using the OmicShare Tools identified potential pathways associated with T2DM, including citrate cycle (TCA cycle), pentose phosphate pathway, primary bile acid biosynthesis, oxidative phosphorylation, arginine and proline metabolism, glycerophospholipid metabolism, arachidonic acid metabolism, pyruvate metabolism, sulfur metabolism, cAMP-signaling pathway, etc. DNL regulates pathways such as citrate cycle (TCA cycle), oxidative phosphorylation, glycerophospholipid metabolism, sulfur metabolism, and other pathways, which are potential pathways for DNL to influence T2DM (Figure 5a,b).

### 2.5. DNL Showed Effects on Serum Proteinic Profiles in db/db Mice with T2DM

**Figure 6:**
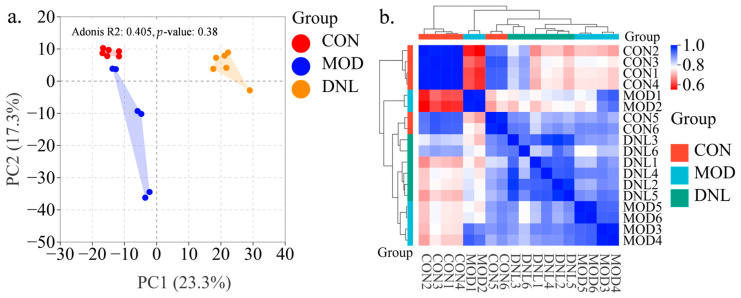
Proteomic profiling reveals systemic modulation by DNL in T2DM mice. (**a**) PCA score plot: Unsupervised multivariate analysis of serum proteomes demonstrated distinct clustering patterns among the control (CON), T2DM model (MOD), and DNL-treated groups. Dimensionality reduction via principal component analysis (PCA) revealed progressive normalization of proteomic profiles in the DNL group, positioning it intermediately between MOD and CON along the first principal component (PC1). (**b**) Hierarchical clustering heatmap: Quantitative proteomic profiling identified group-specific proteomic signatures. The MOD group exhibited disrupted protein correlation networks (blue clusters), while DNL intervention partially restored physiological interaction patterns (red clusters), highlighting its regulatory effects on insulin signaling and inflammatory pathways. Abbreviations: CON, control group; MOD, T2DM model group; DNL, *Dendrobium nobile* Lindl.-treated group.

To improve our understanding of the potential mechanisms of T2DM and the mechanism of action of DNL, we performed non-targeted proteomics analysis of serum samples from the CON group, the MOD group, and the DNL group. As shown in Figure 6a, the PCA plot showed that the CON group was well separated from the MOD group, and the DNL group had a tendency to deviate from the MOD group and approach the CON group. The correlation clustering analysis showed a clear trend of clustering among the three groups, with the DNL group intermediate between the CON group and the MOD group (Figure 6b).

### 2.6. Proteins Changes of DNL on db/db Mice with T2DM

**Figure 7:**
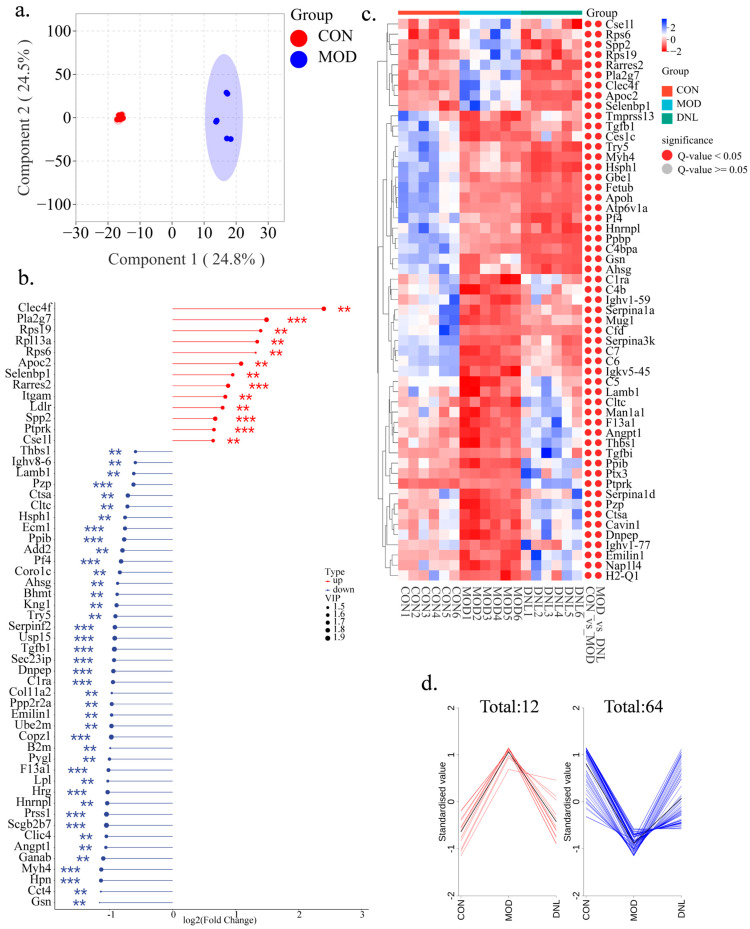
Proteomic regulation by DNL in *db*/*db* mice with T2DM. (**a**). OPLS-DA score plot: Supervised multivariate analysis revealing variable importance in projection (VIP) scores, identifying discriminant proteins between control (CON) and model (MOD) groups. (**b**). VIP-FC lollipop plot: Multiparametric biomarker profiling integrating VIP values, fold change (FC), and statistical significance (*p*-values) of differential proteins (Ranked in the top 55): ** *p* < 0.01, *** *p* < 0.001. (**c**). Hierarchical clustering heatmap: Color-coded visualization of protein expression patterns across CON, MOD, and DNL groups, with annotations for significant differences in CON_VS_MOD and MOD_VS_DNL comparisons. (**d**). *K*-means cluster analysis: Bipartite classification of upregulated/downregulated proteins in CON/MOD groups, demonstrating recovery trends in the DNL intervention group.

The endogenous protein changes in the serum of *db*/*db* mice with T2DM upon DNL intervention were further investigated. The OPLS-DA analysis was performed to identify proteins contributing to the separation of the CON group and the MOD group. The score plot showed a clear separation between the two groups, and cross-validation plots generated from 200 alignment tests showed intercepts of R2 = 0.994 and Q2 = 0.913, indicating good reliability and predictability of the respective models (Figure 7a). Filtering the initial selection of proteins according to the criteria of FC > 1.5, *p* < 0.05, we screened to obtain 113 proteins, of which 13 were upregulated and 100 were downregulated in the MOD group (Figure 7b and Figure S2). The correlation analysis showed that differential proteins with similar abundance clustered together, representing an internal relationship between differential proteins (Figure S3).

The hierarchical clustering dendrogram showed that the proteins in the MOD group were significantly separated from both the CON group and DNL group, while the proteins in the CON group and DNL group had a similar trend. And 55 differential proteins were significantly recovered in the DNL group compared to the MOD group (Figure 7c). Further, the *K*-means clustering analysis showed that DNL recovered 76 differential proteins (64 upregulated and 12 downregulated) (Figure 7d).

### 2.7. DNL Exerted Influences on db/db Mice with T2DM-Related Pathways

**Figure 8:**
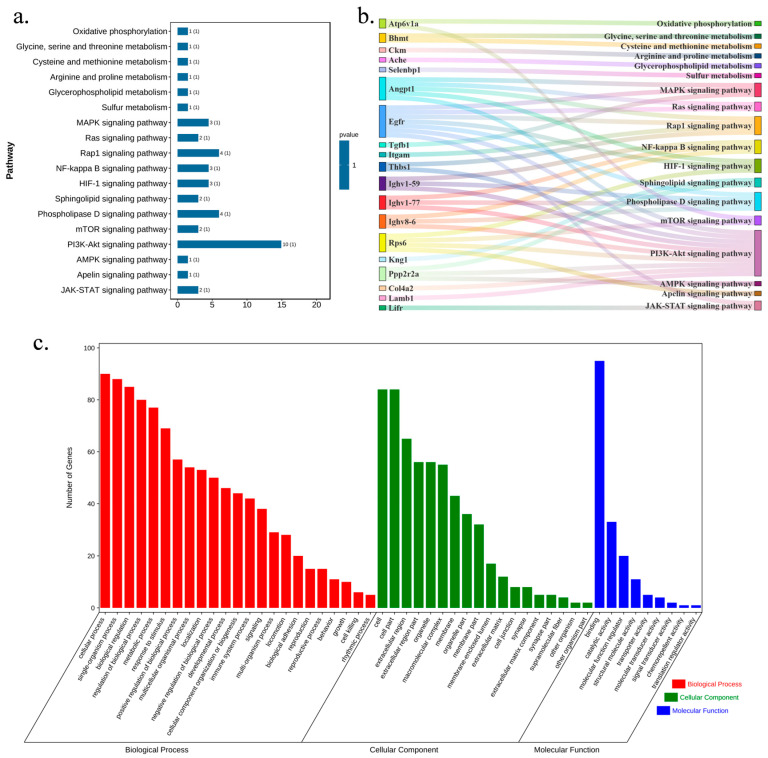
Multi-dimensional pathway mapping of DNL’s therapeutic actions in T2DM mice. (**a**). The KEGG-enrichment bar chart: The enrichment results of differential protein pathways were displayed. (**b**). The sankey diagram: The differential proteins enriched in each pathway were shown. (**c**). The GO classification histogram: Based on the GO classification results, a classification histogram was generated to visually display the GO classification results of differential proteins.

Based on the KEGG database, the enrichment analyses using the OmicShare Tools identified potential pathways associated with T2DM, including oxidative phosphorylation, arginine and proline metabolism, sulfur metabolism, the MAPK-signaling pathway, NF-kappa B-signaling pathway, HIF-1-signaling pathway, mTOR-signaling pathway, PI3K-Akt-signaling pathway, AMPK-signaling pathway, JAK-STAT-signaling pathway, etc. DNL regulated arginine and proline metabolism, sulfur metabolism, the MAPK-signaling pathway, NF-kappa B-signaling pathway, HIF-1-signaling pathway, PI3K-Akt-signaling pathway, and JAK-STAT-signaling pathway, which served as potential pathways for DNL to influence T2DM (Figure 8a,b).

The classification was performed according to GO annotations: biological process (BP), cellular component (CC), and molecular function (MF). The results showed that differential proteins in the MOD group were significantly enriched in several biological processes, molecular functions, and cellular component categories compared to the CON group (*p* < 0.05). Differential proteins from the MOD group were involved in the regulation of metabolic processes, cellular process regulation, signalling, and nitrogen compound metabolism processes. These differential proteins were associated with the regulation of molecular functions, such as catalytic activity, regulators of molecular functions, structural molecule activity, transporter activity, etc. The main cellular components enriched in the MOD group were extracellular regions, organelles, macromolecular complexes, and membranes (Figure 8c).

### 2.8. Metabolomics with Proteomics Revealed Mechanism of DNL Intervention in db/db Mice

**Figure 9:**
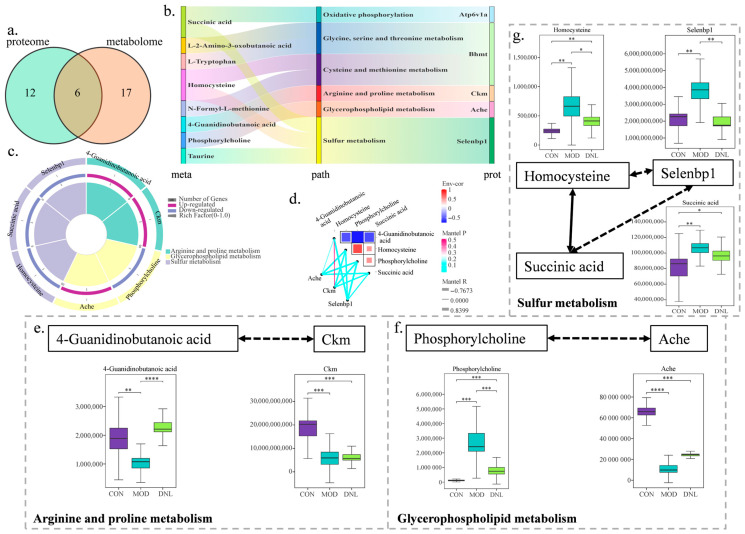
Multi-omics convergence deciphers DNL’s metabolic regulatory network in T2DM mice. (**a**) KEGG pathway intersection: Venn diagram identifies co-enriched pathways (q < 0.05) from metabolomic (blue) and proteomic (yellow) datasets, including oxidative phosphorylation and sulfur metabolism. (**b**) Cross-omics pathway mapping: Sankey diagram visualizes 32 metabolites (left) and 55 proteins (right) converging on arginine/proline metabolism (node width reflects enrichment significance). (**c**) Functional convergence landscape: Circos plot integrates DNL-regulated pathways (outer ring) with corresponding metabolites (green arcs) and proteins (purple arcs), highlighting glycerophospholipid metabolism as the central hub. (**d**) Metabolite–protein interactome: Network heatmap reveals strong positive correlations (r > 0.7, red edges) between 4-guanidinobutanoic acid (metabolite) and Ckm (protein), alongside NF-κB pathway proteins (IκBα, p65) negatively correlating with homocysteine (r < −0.6, blue edges). Pathway-specific mechanistic insights: (**e**) Arginine/proline metabolism: DNL upregulated 4-guanidinobutanoic acid (**** *p* < 0.0001 vs. MOD) and muscle creatine kinase (Ckm), restoring ATP regeneration (solid arrows: enzymatic reactions; dashed arrows: regulatory interactions). (**f**) Glycerophospholipid metabolism: DNL reduced phosphorylcholine ((*** *p* < 0.001) and acetylcholinesterase (Ache), mitigating membrane lipid peroxidation (solid lines: metabolic flux; dashed lines: inhibitory effects). (**g**) Sulfur metabolism: Intervention decreased homocysteine (* *p* < 0.05) and selenium-binding protein 1 (Selenbp1, ** *p* < 0.01) while modulating succinate-mediated inflammatory signaling (solid lines: direct catalysis; dashed lines: allosteric regulation). Abbreviations: CON, control; MOD, T2DM model; DNL, *Dendrobium nobile* Lindl.-treated. Statistical significance: * *p* < 0.05, ** *p* < 0.01, *** *p* < 0.001, **** *p* < 0.0001.

Through the KEGG database, as shown in Figure 9a,b, differential proteins and differential metabolites co-regulated six pathways, including oxidative phosphorylation, glycine, serine and threonine metabolism, cysteine and methionine metabolism, arginine and proline metabolism, glycerophospholipid metabolism, and sulfur metabolism. Among them, DNL regulated three differential proteins and four differential metabolites, which were involved in arginine and proline metabolism, glycerophospholipid metabolism, and sulfur metabolism (Figure 9c). The results of the correlation analysis showed (Table S4, Figure 9d) that Ache had a low correlation (0.2–0.39) with 4-guanidinobutanoic acid, ache had moderate correlation (0.4–0.69) with homocysteine and succinic acid, and Ache had a high correlation (0.7–0.89) with phosphorylcholine; Ckm had an extremely low correlation (0–0.19) with 4-Guanidinobutanoic acid, Ckm had moderate correlation (0.4–0.69) with homocysteine, phosphorylcholine and succinic acid; Selenbp1 was moderately correlated with succinic acid and homocysteine (0.4–0.69), and Selenbp1 was highly correlated with 4-Guanidinobutanoic acid and phosphorylcholine (0.7–0.89). In arginine and proline metabolism, DNL significantly modulated the levels of 4-Guanidinobutanoic acid (*p* < 0.001); in glycerophospholipid metabolism, DNL significantly modulated the level of phosphorylcholine (*p* < 0.001); in sulfur metabolism, DNL significantly modulated the levels of Homocysteine (*p* < 0.05) and Selenbp1 (*p* < 0.01) (Figure 9e–g).

## 3. Discussion

In this study, we constructed a T2DM model using *db*/*db* mice. Thirty-six days later, mice in the MOD group spontaneously developed T2DM symptoms, such as elevated body weight, FBG, HbA1c, and insulin levels. In addition, mice in the MOD group had a significantly lower kidney index and significantly higher serum levels of Cr, BUN, and MDA. H&E staining showed that the kidney tissues of mice in the MOD group were diseased. We used DNL to intervene in *db*/*db* mice for 36 days, and all the above symptoms were significantly improved, which indicated that DNL administration had significant preventive and therapeutic effects on T2DM.

The mechanism of DNL intervention in the *db*/*db* mouse model was subsequently explored through the integration of proteomics and metabolomics. Differentially expressed proteins and metabolites were identified, elucidating the mechanisms of DNL intervention in T2DM. Serum samples from each group clustered closely in PCA score plots. Proteins and metabolites from the MOD group were separated from the CON group and DNL group, and the DNL group was closer to the CON group. The correlation clustering plot showed that the proteins and metabolites of the MOD group were separated from the CON group and DNL group, and the DNL group was closer to the CON group. This suggested that the metabolic profile of the *db*/*db* mice was altered, and that intervention with DNL resulted in a similar metabolic profile to that of the CON group of mice. By KEGG-enrichment analysis, DNL modulated three pathways co-enriched by metabolomics and proteomics in *db*/*db* mice and with altered protein and metabolite content. These pathways included arginine and proline metabolism, glycerophospholipid metabolism, and sulfur metabolism. DNL regulated differentially expressed proteins such as Ckm, Ache, and Selenbp1 and differentially expressed metabolites such as 4-Guanidinobutanoic acid, phosphorylcholine, homocysteine, and succinic acid.

Arginine and proline metabolism regulate the balance between ROS, as well as NO production and clearance. Its disturbance accelerates T2DM pathology by synergistically promoting inflammatory response and oxidative damage, disrupting energy metabolism and insulin sensitivity [17,18]. Further, 4-Guanidinobutyric acid is a common arginine metabolite involved in the metabolism of arginine and proline (creatinine pathway), which is essential for energy metabolism [19,20]. In the present study, it was detected that the level of 4-guanidinobutyric acid is lower in the MOD group than in the CON group, and DNL-mediated upregulation of 4-guanidinobutyric acid suggests enhanced arginine/proline metabolism, which may contribute to the therapeutic effect. Ckm maintains the rapid regeneration of ATP in the muscle through the creatine–phosphocreatine cycle, which provides energy for muscle contraction and metabolism. In patients with T2DM, metabolic disturbances lead to a decrease in Ckm activity, which impairs the muscle’s ability to respond rapidly to energy demands [21]. In the present study, the level of Ckm was found to be lower in the MOD group than in the CON group, and DNL significantly increased the level of Ckm.

Glycerophospholipid metabolism directly affects the ratio and composition of cell membrane lipids. In patients with T2DM, an imbalance in the ratio of PC/PE in the cell membrane reduces membrane fluidity and impairs the efficiency of insulin receptor binding to insulin, leading to insulin resistance [22]. Glycerophospholipid metabolism is also critical for maintaining pancreatic β-cell membrane integrity and function. Metabolic disorders may lead to cell membrane disruption and increased lipotoxicity, triggering β-cell apoptosis or dysfunction and reducing insulin secretion [23]. Phosphocholine is an important intermediate product of phospholipid metabolism and is widely involved in biological processes, such as cell membrane structure, signal transduction, and regulation of inflammatory responses [24]. Increased phosphocholine deteriorates insulin signalling and causes insulin resistance [25]. Blocking the synthesis of lysophosphatidic acid from phosphocholine restores serum GLP-1 levels, insulin secretion, and glucose tolerance [26]. DNL significantly reduced the level of phosphocholine and suppressed insulin resistance and inflammation in *db*/*db* mice with T2DM.

Sulfur metabolism involves a wide range of biochemical reactions of elemental sulfur and is an important process in maintaining life activities. Sulfur metabolism is involved in the regulation of antioxidant defence, inflammation regulation, energy metabolism, and pancreatic function and is closely related to the development of T2DM [27]. Homocysteine is an intermediate product of methionine metabolism, and its metabolism is dependent on folate, vitamin B6, and vitamin B12. When abnormal metabolism leads to homocysteine accumulation, it can directly interfere with the insulin-signaling pathway [28]. It has been shown that homocysteine induces insulin resistance and contributes to the diabetic phenotype through protein cysteine–homocysteinylation of the pre-insulin receptor [29]. Homocysteine can exacerbate the chronic inflammatory state by activating the NF-κB pathway and exacerbating the release of pro-inflammatory cytokines (e.g., IL-6, TNF-α) [30]. Homocysteine also induces the activation of pro-inflammatory macrophages (M1 type) and inhibits the function of anti-inflammatory macrophages (M2 type), which further worsens the inflammatory microenvironment and reduces insulin sensitivity [31]. Succinic acid can act as a ligand to activate SUCNR1 and enhance insulin secretion. Prolonged increased levels of succinic acid may lead to the overactivation of the receptor, which in turn triggers β-cell dysfunction and affects normal insulin secretion [32]. In macrophages, succinic acid activates inflammatory pathways and increases the release of pro-inflammatory cytokines, leading to chronic low-grade inflammation [33]. Up-regulation of SELENBP1 expression inhibits the activation of PI3K/AKT/mTOR signaling pathway, thereby hindering the malignant development of lung adenocarcinoma. [34], which attenuates insulin-stimulated glucose uptake and promotes the development of insulin resistance [35]. In this study, the levels of homocysteine, succinic acid, and Selenbp1 were detected to be significantly higher in the MOD group than in the CON group, and DNL significantly reduced the levels of cysteine, succinic acid, and Selenbp1, exerting anti-inflammatory, anti-insulin resistance, and alleviating T2DM symptoms.

This study focuses on the overall intervention mechanism of DNL. The existing literature indicates that the primary active compounds in DNL include alkaloids (such as dendrobine), polysaccharides, and flavonoids [6]. Dendrobine compounds can promote mitochondrial function and inhibit oxidative phosphorylation [7]. This study observed that DNL significantly upregulates 4-guanidino butyric acid (a key intermediate in arginine/proline metabolism) and creatine kinase (CKM), which may be partially attributed to the alkaloids’ reprogramming effect on energy metabolism. This explains the restoration of ATP regeneration capacity after DNL intervention, alleviating energy imbalance in T2DM (Figure 9e). Dendrobium polysaccharides have been shown to activate the PI3K/Akt- and AMPK-signalling pathways, improving glucose uptake in liver and muscle tissues [8]. This aligns closely with the findings in this study, where DNL inhibits the PI3K-Akt pathway (by downregulating Selenbp1) and regulates glycerophospholipid metabolism (Figure 9f,g). The flavonoid components in DNL (such as naringin and kaempferol) possess the ability to inhibit the NF-κB pathway and scavenge ROS [36]. This directly supports the findings of this study: DNL blocks the NF-κB inflammatory cascade by downregulating homocysteine and succinate levels (Figure 9g) and reduces oxidative stress markers such as MDA (Figure 2h).

## 4. Materials and Methods

### 4.1. Animal Experiments

Male *db*/*db* and *db*/m mice (seven-week-old, SPF-grade) were obtained from GemPharmatech Co., Ltd. (Nanjing, China). The experimental protocol was designed with strict reference to relevant ethical regulations and was approved by the Ethics Committee of Hainan Medical College. This experiment had four groups: the control (CON) group, the model (MOD) group, the metformin hydrochloride (MET) group, and the *Dendrobium nobile* Lindl. (DNL) group, which were fed normal diets. The mice in each treatment group were provided MET (65.0 mg/kg/d) and DNL (10 g/kg/d) for 4 weeks (n = 12). The MET was purchased from Merck Pharmaceutical (Nantong, China) Co. The study protocols were ethically reviewed and approved by the Institutional Review Board of Hainan Medical University (Ethics Approval Number: HYLL2024-5-09, approved on 31 May 2024).

### 4.2. DNL Preparation

DNL was prepared according to ≪Pharmacopoeia of the People’s Republic of China (2020 Edition)≫ [37]. 50 g of dried DNL herbs (Hainan Shengrong Biotechnology Co., Ltd., Haikou, China) were weighed, soaked in 400 mL of water for 1 h, decocted for 30 min, replenished with 400 mL of water, and decocted for another 30 min to obtain 150 mL of drug solution. Subsequent rotary evaporation was conducted using a rotary evaporator (HEI-VAP CORE ML, Heidolph, Schwabach, Germany) (150 rpm; 70 °C; 1.5 h) to obtain 30 mL of DNL herb concentrate and further made into freeze-dried powder.

### 4.3. General Status Observation and Monitoring of Indicators

During the experimental period, mice were weighed weekly and observed for mental status, reactivity, activity, changes in hair colour, and feeding. Tail vein blood was taken every week, and FBG was measured by GA-3 blood glucose meter (Sanuo Biosensing (Changsha, China) Co., Ltd.). Tail vein blood was taken 1 day before the end of the experiment, and HbA1c was measured by glycated haemoglobin kit (Sanuo Biosensing (Changsha, China) Co., Ltd.).

### 4.4. Histopathological Examination

Kidney tissues were fixed with 4% paraformaldehyde (PFA) and then embedded in paraffin. Specimens were sectioned at 4 μm thickness and stained with haematoxylin and eosin (H&E). Photographs of random areas of the stained sections were taken using a light microscope (Leica Microsystems, Wetzlar, Germany).

### 4.5. Serum Biochemical Analysis

Serum levels of creatinine (Cr), urea nitrogen (BUN), and malondialdehyde (MDA) in *db*/*db* mice were determined using enzyme-linked immunosorbent kits (Nanjing Jiancheng Institute of Bioengineering, Nanjing, China) according to the manufacturer’s guidelines.

### 4.6. Sample Preparation for Metabolomics Analysis

The serums were centrifuged for 10 min (12,000 rpm, 4 °C). The supernatants were transferred to 2 mL centrifuge tubes, concentrated, and dried. The samples dissolved in 200 μL of 50% acetonitrile solution (2-chloro-1-phenylalanine (4 ppm)) were filtered with a 0.22 μm filter membrane and transferred to a detection bottle for LC-MS detection.

### 4.7. Liquid Chromatographic and Mass Spectrometry Conditions

Metabolomics analysis was performed using the Vanquish-Liquid chromatograph (LC) system coupled with the Orbitrap Exploris 120 mass spectrometer in both positive and negative ionization modes. Chromatographic conditions for LC analysis of metabolomics: flow speed: 0.3 mL/min; column temperature:40 °C; injection volume: 2 μL. Mass spectrometry conditions of metabolomics: spray voltage(+): 3.50 kV; spray voltage(−): −2.50 kV; sheath gas pressure: 40 arb; aux gas flow: 10 arb; capillary temperature: 325 °C; MS1 resolution: 60,000 FWHM; MS1 range: *m*/*z* 100~1000; normalized collision energy: 30%; MS/MS resolution: 15,000 FWHM; dynamic exclusion time: automatic. Detailed positive/negative ion mode gradient elution program for LC analysis was provided in Tables S1 and S2.

### 4.8. Metabolomics Data Processing

The principal component analysis (PCA) and orthogonal partial least-squares discriminant analysis (OPLS-DA) were used to build the models by the metware cloud (https://cloud.metware.cn/ (accessed on 15–16 December 2024)). Finally, statistically significant metabolites were defined as those with *p* values < 0.05 and VIP values > 1. Differential metabolites were subjected to a pathway analysis using the OmicShare Tools (https://www.omicshare.com/ (accessed on 17 December 2024)). Metabolomics-discovered compounds were then linked to KEGG pathways for biological interpretation of higher-level system function.

### 4.9. Proteomics Analysis of Sample Preparation

Protein samples were incubated with 5 mM DTT at 37 °C for 1 h and then returned to room temperature. Then, 10 mM of iodoacetamide were added and incubated for 45 min at room temperature without light, and the sample was diluted four times with 25 mM of ammonium bicarbonate, while trypsin was added according to the ratio of protein to trypsin 50:1 and incubated overnight at 37 °C. The next day, formic acid was added to adjust the pH to less than 3, and then the enzyme digestion was terminated. The samples were desalted using a C18 desalting column, activated with 100% acetonitrile, equilibrated with 0.1% formic acid, loaded onto the column, washed with 0.1% formic acid to remove impurities, and finally eluted with 70% acetonitrile, and the flow-through solution was collected and lyophilised.

### 4.10. Chromatographic and Mass Spectrometry Conditions

Proteomics analysis was performed using the RIGOL L-3000 High Performance Liquid Chromatography System (RIGOL TECHNOLOGIES Co., Ltd., Suzhou, China) coupled with the Orbitrap Exploris^TM^ 480 mass spectrometer (Thermo Fisher Scientific, Waltham, MA, USA). Mass spectrometry conditions of proteomics: compensation voltage: −45/−65·s^−1^; ion spray voltage: 2.0 kV; ion transmission tube temperature: 320 °C; full scanning range of mass spectrometry: *m*/*z* 350–1500; primary mass spectral resolution: 120,000 (*m*/*z*); automatic gain control: 4 × 10^5^; maximum injection time: 50 ms; secondary mass spectral resolution: 15,000 (200 *m*/*z*); automatic gain control: 5 × 10^4^; maximum injection time: 22 ms; and peptide fragmentation collision energy: 33%. The gradient elution program for LC analysis of proteomics was detailed and provided in Table S3.

### 4.11. Proteomics Data Processing

The principal component analysis (PCA) and orthogonal partial least-squares discriminant analysis (OPLS-DA) were used to build the models by the Metware Cloud (https://cloud.metware.cn/). Finally, statistically significant proteins were defined as those with *p* values < 0.05 and FC > 1.5. Differential proteins were subjected to a pathway analysis using the OmicShare Tools (https://www.omicshare.com/). Metabolomics-discovered compounds were then linked to KEGG pathways and GO enrichments for biological interpretation of higher-level system function.

### 4.12. Statistical Analysis

Student’s *t*-test was performed using Origin version 2021 software for statistical comparisons. Statistical significance was determined at a threshold of *p* < 0.05, thus delineating significant differences among the groups being compared.

## 5. Conclusions

This study systematically elucidated the anti-diabetic mechanisms of DNL through integrated metabolomics and proteomics in a *db*/*db* mouse model of T2D. DNL intervention significantly ameliorated hyperglycemia, insulin resistance, and renal dysfunction, restoring metabolic homeostasis and alleviating pathological phenotypes. Multi-omics analysis identified 39 differential metabolites and 113 differentially expressed proteins, with integrative pathway mapping highlighting DNL’s regulation of arginine/proline metabolism, glycerophospholipid metabolism, and sulfur metabolism. Mechanistically, DNL suppressed oxidative stress and inflammation by modulating homocysteine and succinic acid levels, restored insulin sensitivity via glycerophospholipid balance, and enhanced energy metabolism through TCA cycle reactivation. The correlation network between metabolites and proteins further validated DNL’s multi-target action, particularly its inhibition of NF-κB and PI3K-Akt pathways linked to insulin resistance. These findings not only decode DNL’s therapeutic potential but also provide a multi-omics framework to probe the systemic mechanisms of traditional herbal interventions. This work provides critical insights for developing DNL-based therapies and underscores the value of integrative omics in bridging traditional medicine with modern pharmacodynamics.

## Data Availability

Data is contained within the article or Supplementary Materials.

## Supplementary Materials

The following supporting information can be downloaded at: https://www.mdpi.com/article/10.3390/ph18071061/s1.
