# Supplementary material for: Integrated Omics Reveal Dendrobium nobile Lindl.’s Anti-Diabetic Mechanisms via Arginine/Proline and Glycerophospholipid Pathways
Source: Pharmaceuticals (Basel). 2025 Jul 18;18(7):1061. doi: 10.3390/ph18071061 (PMC12300346; doi:10.3390/ph18071061)
Supplement: Supplementary file 1 [file pharmaceuticals-18-01061-s001.zip › pharmaceuticals-3699732-supplementary.pdf]

Table S1. Positive ion mode gradient elution program table for LC analysis of metabolomics.

Time (min)	Flow speed (ml/min)	A2 % (0.1 % formic acid water)	B2 % (0.1 % formic acid acetonitrile)
0	0.3	90	10
1	0.3	90	10
5	0.3	2	98
6.5	0.3	2	98
6.6	0.3	90	10
8	0.3	90	10

Table S2. Negative ion mode gradient elution program table for LC analysis of metabolomics.

Time (min)	Flow speed (ml/min)	A3 % (5 mM ammonium formate water)	B3 % (acetonitrile)
0	0.3	90	10
1	0.3	90	10
5	0.3	2	98
6.5	0.3	2	98
6.6	0.3	90	10
8	0.3	90	10

Table S3. The gradient elution program table for LC analysis of proteomics.

Time (min)	A2 % (100% water (0.1 % formic acid))	B2 % (80% acetonitrile (0.1 % formic acid))
0	94	6
8	94	6
13	88	12
46	70	30
53	60	40
54	5	95
64	5	95
65	94	6

Table S4. Correlation coefficients between three differential proteins and four differential metabolites regulated by DNL

Protein	metabolite	cor	p_value
Ache	4-Guanidinobutanoic acid	0.339065	0.168685
Ache	Homocysteine	-0.64877	0.003582
Ache	Phosphorylcholine	-0.76733	0.000202
Ache	Succinic acid	-0.59984	0.008501
Ckm	4-Guanidinobutanoic acid	0.162639	0.51906
Ckm	Homocysteine	-0.58848	0.010197
Ckm	Phosphorylcholine	-0.65403	0.003236
Ckm	Succinic acid	-0.55154	0.017656
Selenbp1	4-Guanidinobutanoic acid	-0.75944	0.000256
Selenbp1	Homocysteine	0.643192	0.003982
Selenbp1	Phosphorylcholine	0.839862	1.30E-05
Selenbp1	Succinic acid	0.414741	0.087018

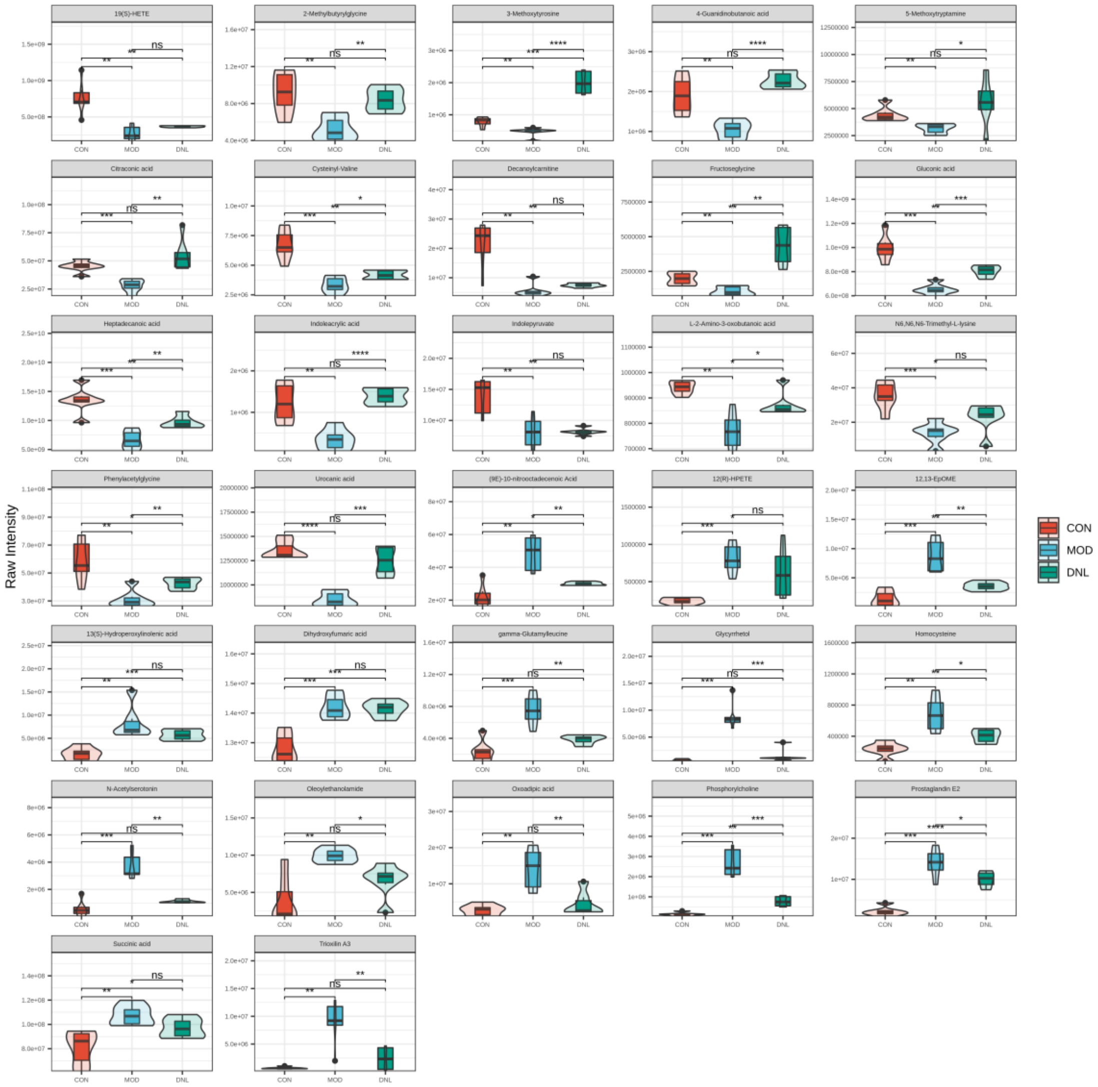

Figure S1. Violin plots: Density distribution visualization illustrating concentration variations of differential metabolites among CON, MOD, and DNL groups. Data are expressed as mean ± SEM. Statistical significance: * $p < 0.05$, ** $p < 0.01$, *** $p < 0.001$ (two-tailed Student's t-test).

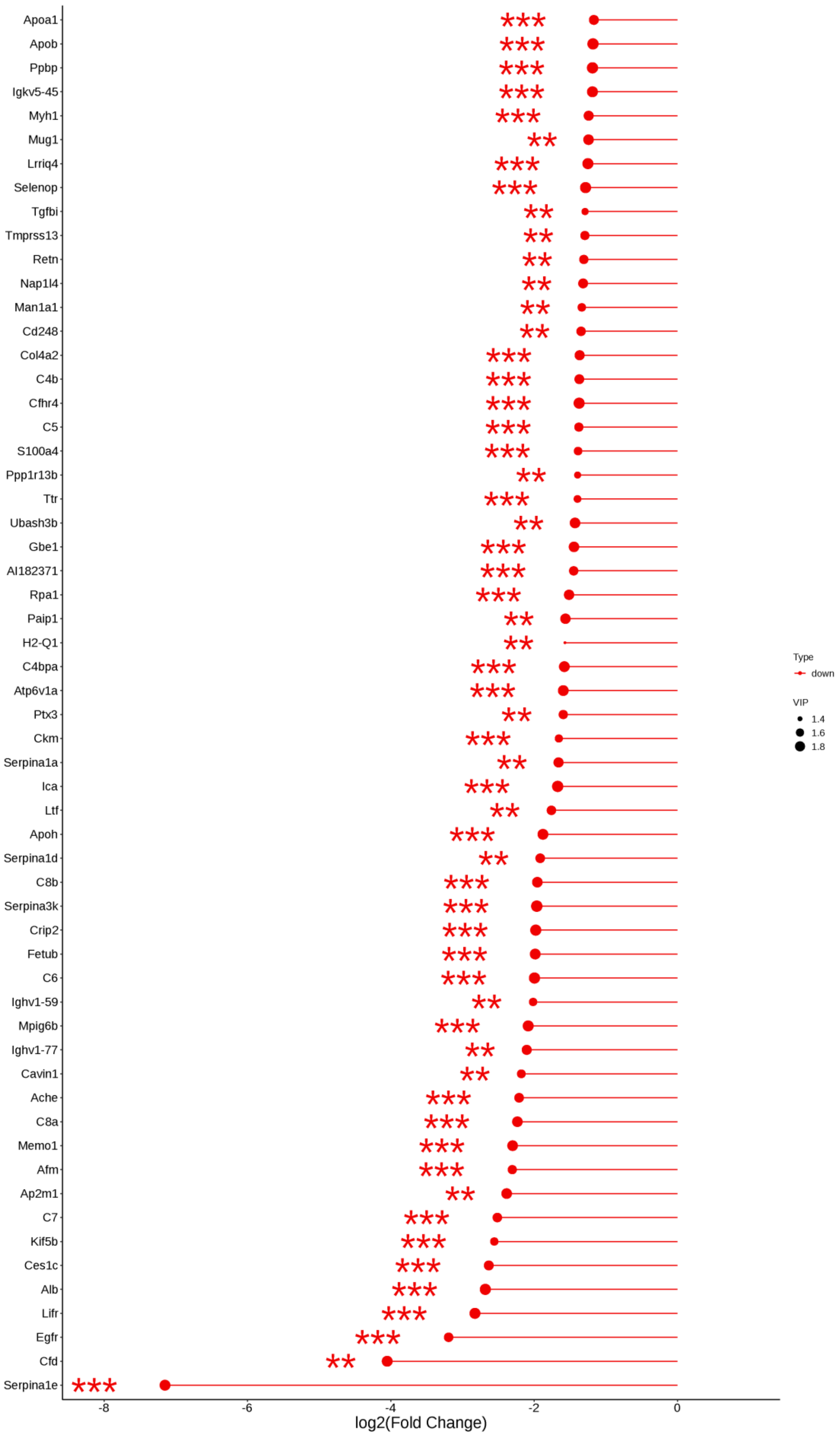
Apoa1
Apob
Ppbp
Igkv5-45
Myh1
Mug1
Lrriq4
Selenop
Tgfbi
Tmprss13
Retn
Nap1l4
Man1a1
Cd248
Col4a2
C4b
Cfhr4
C5
S100a4
Ppp1r13b
Ttr
Ubash3b
Gbe1
AI182371
Rpa1
Paip1
H2-Q1
C4bpa
Atp6v1a
Ptx3
Ckm
Serpina1a
Ica
Ltf
Apoh
Serpina1d
C8b
Serpina3k
Crip2
Fetub
C6
Ighv1-59
Mpig6b
Ighv1-77
Cavin1
Ache
C8a
Memo1
Afm
Ap2m1
C7
Kif5b
Ces1c
Alb
Lifr
Egfr
Cfd
Serpina1e
-8
-6
-4
-2
0
log2(Fold Change)
Type
down
VIP
1.4
1.6
1.8

Figure S2. VIP-FC lollipop plot: Multiparametric biomarker profiling integrating VIP values, fold change (FC), and statistical significance (p-values) of differential proteins (The remaining 58 out of 113): * $p < 0.05$, ** $p < 0.01$, *** $p < 0.001$.

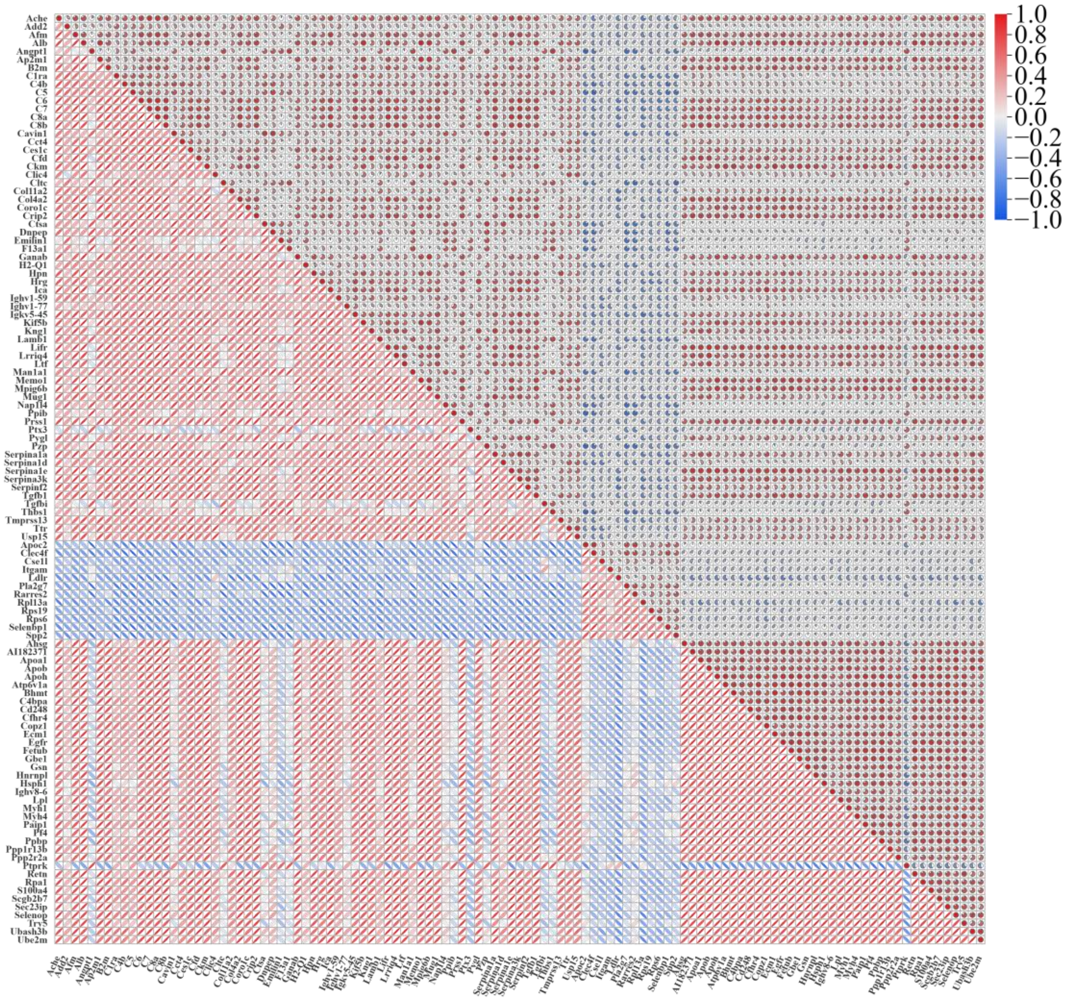

Figure S3. Correlation heatmap: Pair-wise correlation matrix of differential proteins based on expression levels in CON and MOD groups, mapping inter-protein interaction networks.
